# NHS patients, staff, and visitor viewpoints of smoking within a hospitals’ ground: a qualitative analysis

**DOI:** 10.1186/1471-2458-14-1015

**Published:** 2014-09-29

**Authors:** Alina Serafin, Sarah Franklin, Rashesh Mehta, Scott Crosby, Diane Lee, Becky Edlin, Bridgette M Bewick

**Affiliations:** Leeds Teaching Hospital NHS Trust, St James’s University Hospital, Leeds, LS9 7TF UK; Wakefield Metropolitan District Council, Wakefield, WF1 2DD UK; Magpie Creative Communications Ltd, Leeds, LS2 9NG UK; Leeds Institute of Health Sciences, School of Medicine, University of Leeds, Leeds, LS2 9LJ UK

## Abstract

**Background:**

Smoking is a public health concern and an avoidable cause of morbidity and mortality. Widening tobacco control policies might help shift social norms, the acceptability of exposing others to second-hand smoke, and cultural attitudes towards smoking. This study explored patient, staff, and visitor viewpoints of smoking within the grounds of a National Health Service hospital.

**Methods:**

Analysis of free text responses given as part of a larger repeat cross sectional questionnaire study. Free text qualitative responses analysed using thematic analysis. Pinderfields Hospital, a UK National Health Service hospital in the county of Yorkshire, provides a health service to around half a million people living in the Wakefield and North Kirklees area. Surveys were distributed 10^th^-18^th^ September and 17^th^-21^st^ December 2012. Of the n=952 participants who completed an anonymous survey n=306 participants provided a response to the optional free text question.

**Results:**

Thematic analysis revealed 5 distinct themes: (1) smoking is a dirty problem; (2) smokers are free to do as they wish; (3) the poor smoker; (4) smoke in our space: the battleground; and (5) no smoking please. Of the n=272 represented by the five themes, generally people accepted that smoking is socially unacceptable but their understanding of smoking behaviours and attitudes towards management and control of smoking differed. There was a strong sense that action is needed to separate the space smokers and non-smokers share. We identified a distinct group of participants that supported a hard line approach and suggested enforcing the no smoking policy through fines and monitoring.

**Conclusions:**

Smoking on hospital grounds remains a contentious issue. Participants acknowledge that smoking is an increasingly unacceptable social behaviour but their understanding and acceptance of smokers vary. There is a strong sense of dislike about the impact of smoke and smokers on the shared hospital environment, with a focus on the hospital entrance. Participants suggest separating smokers and non-smokers and moving smokers away from the hospital entrance with the introduction of smoking shelters. These results suggest a complex narrative that should be investigated further to inform the implementation of the no-smoking policy across hospital settings.

**Electronic supplementary material:**

The online version of this article (doi:10.1186/1471-2458-14-1015) contains supplementary material, which is available to authorized users.

## Background

Smoking is a major public health concern and an avoidable cause of morbidity and mortality in the United Kingdom [1]. The prevalence rate of smoking in England is 20% in the adult population [2]. Between 2011/12, there were 1.6 million hospital admissions in adults over 35 years of age with a primary diagnosis of a disease that can be caused by smoking [2]. Nationally the cost of smoking to the NHS has been calculated to be between £2.7 billion [3] and £5.2 billion [4].

Smokefree legislation was introduced in England on 1^st^ July 2007 with the Health Act 2006 banning smoking in all enclosed and substantially enclosed work and public places. Evidence suggests that smoke-free legislation has been effective in protecting people from the harmful effects of second-hand smoke exposure while in public places [5, 6, 2]. In addition, there have been public health benefits in reducing cigarette consumption and prevalence [3] and potentially changing attitudes towards smoking.

The national smoking ban inside buildings is widely understood and adhered to [3]. However there are now concerns that staff, patients and visitors at our National Health Service (NHS) hospitals have relocated to smoke at hospital entrances and on hospital grounds- despite a no smoking policy [7]. This behaviour is increasingly becoming a public health concern [7]. Widening tobacco control policies might help shift social norms about the acceptability of exposing others to second-hand smoke and change cultural attitudes and norms towards smoking. There is a lack of evidence however about how much people support smokefree policies in health related environments.

This study, through analysis of free text responses given as part of a larger repeat cross sectional questionnaire study, aims to explore hospital user perceptions and viewpoints of smoking in and around a UK NHS hospital.

## Method

### Design

The current qualitative study is a secondary analysis of free text responses provided by participants to an optional question included as part of a wider questionnaire investigating the attitudes and behaviour of hospital patients, staff, and visitors towards smoking on hospital premises. [Franklin, Crosby, Lee, Nehta, Edlin, Bewick:The impact of the social norms approach campaign on reducing levels of misperceptions around smokefree hospital entrances amongst patients, staff, and visitors of a NHS hospital: Before and after cross-sectional design, submitted]. The original study utilised two cross-sectional survey data collection points and aimed to investigate actual and perceived social norms associated with smoking in hospital entrances and on hospital grounds.

### Setting

Pinderfields Hospital is a UK National Health Service hospital based in Wakefield in the county of Yorkshire. It is part of the Mid Yorkshire Hospitals NHS Trust which provides community, acute (hospital-based treatment) and specialist health services to around half a million people living in the Wakefield and North Kirklees area. Surveys were distributed between 10-18^th^ September and 17^th^-21^st^ December 2012.

### Participants

All hospital staff, patients and visitors on the premises of Pinderfields hospital during data collection periods were eligible to participate. Surveys were collected from n = 481 participants during September (n = 164 patients, n = 143 hospital visitors, n = 163 hospital staff, n = 11 unknown/other) and n = 459 participants during December (n = 157 patients, n = 143 hospital visitors, n = 156 hospital staff, n = 3 unknown/other).

### Ethics

Research and Development approval was received (Mid Yorkshire NHS Trust). The study was approved as an audit and evaluation of smoking behaviour on Pinderfields hospital grounds; R&D Permissions were granted via NHS Wakefield.

All participants were provided with information on the audit to enable them to make an informed decision as to whether to participate in the audit. Consent was deemed to have been given if individuals completed and returned the pen and paper survey. Individuals had the right to refuse to participate; due to the anonymous completion of the survey once surveys were returned it was not possible for data to be withdrawn.

In accordance with BioMed Central editorial policies for reporting qualitative studies. this article adheres to the RATS guideline (see Additional file 1).

### Procedure

Convenience sampling was undertaken, and data collection was organised to ensure that all accessible areas of the hospital building and grounds were covered. Paper surveys were distributed by employees of Magpie Creative Communications throughout the hospital and grounds (e.g. hospital wards, administrative staff areas, canteen areas, hospital shuttle bus queue). An incentive for the completion and return of each survey was given to respondents being a donation of £1 for a local charity paid on their behalf. Three choices of charity were given: The Mid Yorkshire Hospitals NHS Trust Charitable Fund, Wakefield Hospice, and Macmillan Nurses.

### Data collection

The wider study collected data on the difference between the perceived and reported levels of support for smokefree hospital entrances and grounds. The survey title explained to participants that the questions were aimed to understand what people think about smoking around the grounds of Pinderfields Hospital. These questions included one free text box that asked “If you have any other comments you would like to add, please put them here”.

### Analysis

The comments from 306 participants were broken down into 480 utterances with separate meanings. Data were analysed using a thematic analysis. Using an inductive approach, utterances were searched for items of interest and recurrent themes. Codes were iteratively developed with discussions between authors AS and BMB; disagreements were resolved through a process of consensus. The final coding structure enabled categorisation of all utterances (Additional file 2).

## Results

In total 1000 surveys were distributed and 963 (96%) were completed, returned, and included in the current analysis. Of these 23 were excluded (n = 20 age < 18 years, n = 3 < 50% completion). Of the 940 participants n = 306 chose to provide a free text response at the end of the survey. The responders reported similar levels of smoking in the hospital entrance and on hospital grounds compared with those who did not provide a free text response but the responder sub-sample had a higher proportion of staff (see Table 1).

**Table 1:** **Comparison of demographic and smoking status characteristics of participants who choose to respond to the optional free text book compared with non-responders**

	Free text response not provided (n = 634)		Free text response provided (n = 306)		All survey participants (n = 940)	
	n	(%)	n	(%)	n	(%)
User group						
Patient	259	(41%)	62	(20%)	321	(34%)
Staff	164	(26%)	155	(51%)	319	(34%)
Visitor	203	(32%)	83	(27%)	286	(30%)
Female	413	(65%)	223	(73%)	636	(68%)
Ethnicity						
White/White British	563	(89%)	283	(93%)	846	(90%)
Asian/Asian British	41	(7%)	11	(4%)	52	( 6%)
Smoking status: Non-smoker	527	(83%)	267	(87%)	794	(86%)
Do not smoke in entrance	619	(98%)	297	(97%)	916	(97%)
Do not smoke on hospital grounds	586	(92%)	281	(92%)	867	(92%)

Analysis revealed 23 codes (see Additional file 2 for list of codes), which were bought together to form 6 themes. Five of the themes speak directly to participants’ views surrounding smoking and smoking on hospital premises. In addition a sixth ‘miscellaneous’ theme was identified. This sixth theme included comments from n = 52 participants; of these n = 34 participants were not represented in any other theme. Miscellaneous comments were those that were: incomplete and therefore meaning was unable to be deciphered (e.g. “*Fri on one end, food at other ..*.”); comments only on the process and/or content of the project (e.g. “*Yet another pointless survey …*”*)*. After reviewing the content of comments included in this theme it was ascertained that the comments held no meaning of relevance to participants views of smoking and/or smoking on hospital premises and these comments were not included in the synthesis of results – this effectively meant that n = 34 participants were not included in further analysis or synthesis of findings.

Thematic analysis revealed 5 distinct themes representing the views of n = 272 participants (n = 52 patients, n = 141 staff, n = 74 visitors, n = 5 other). The five themes were: (1) smoking is a dirty problem; (2) smokers are free to do as they wish; (3) the poor smoker; (4) smoke in our space: the battleground; and (5) no smoking please.

In total 185 (68%) of the 272 participants have comments located in only one theme. Individual themes have between 37% and 63% of comments expressed by participants whose comments were encapsulated in one theme only. From the comments it is apparent that some individuals hold values and express views that span across more than one theme. The percentage of the n = 272 who expressed views consistent with each of the themes is provided alongside the number of participants from each user group who had comments included in each theme; this percentage provides an indication of the distribution of viewpoints across the current sample.

### Theme 1: smoking is a dirty problem

Utterances revealed that hospital users view smoking as a harmful and dirty habit that impacts upon non-smokers. The act of smoking near hospitals goes against the idea that hospitals are places of health and wellbeing, and it offended people to see other people smoking around them. This theme encapsulates negative thoughts and associations with smoking from n = 71 (26%; n = 18 patients, n = 41 staff, n = 12 visitors) participants.

Participants commented on the unpleasant sight of cigarette butts on the ground and the smell of smoke.

*“Hospital entrances look unkempt…with cigarette butts all over the floor”*

They voiced dislike or disgust for smokers or the effects of smoking.

*“I think it is disgusting that people are continuing to smoke”*

There was also an opinion that smoking is dirty, and this was linked to ideas of infection. Patients in particular were depicted as being the bearer of infection when they leave the hospital building to smoke and then return.

*“patients who smoke then enter wards are an infection control risk”*

Participants commented on the negative association of seeing people smoking in or around the hospital site. There was a judgement that smokers should not smoke near a hospital as it does not send the right message.

*“Where the smokers congregate outside the main entrances gives an extremely bad impression of this hospital trust”*

Some participants felt that their rights as non-smokers were being infringed upon due to the effects of second hand smoke, and expressed a view that there is a need for change or control. However they did not go further to elucidate exactly what should be done to control or change smoking behaviour.

*“being a non smoker I feel my right to breathe smoke free air is compromised”*

Values emerged of an accepted social norm that certain individuals, such as patients and in particular pregnant women, should not smoke. People expressed that seeing these people smoke within hospital grounds, a space which should promote well-being, is not appropriate.

*“People are here to get well, not add to their illnesses”*

Smoking was seen as a socially unacceptable practice that should not happen within an environment that promotes health and well-being.

### Theme 2: smokers are free to do as they wish

This theme contains utterances from n = 12 (4%; n = 3 patients, n = 5 staff, n = 4 visitors) participants, who expressed judgements that smokers have a right to do as they wish. Participants conveyed a view that individuals have the freedom to choose how to behave.

*“It is 100% legal to smoke outside”*

*“everyone has a choice and if they choose to smoke they should be allowed”*

The utterances within this theme predominately voiced an opinion that it is not appropriate to become involved in attempting to change another person’s smoking behaviour. Out of the 12 participants represented in this theme, 42% (n = 5) did not make any other comments. However, 58% (n = 7) of participants coupled this view with comments that span the remaining 4 themes. Half (n = 6) suggested a smoking shelter to separate smokers from other people.

### Theme 3: The poor smoker

Participants (20%, n = 54; n = 10 patients, n = 25 staff, n = 16 visitors, n = 3 other) acknowledged that although people should not smoke there are some that continue to do so. Utterances revealed how participants attempted to explain why people may smoke, and mentioned “addiction” and “stress” as reasons.

*“I respect how difficult it is for smokers…in a hospital some people may be experiencing a great deal of stress and need a cigarette”*

There is a feeling of trying to help smokers, by helping them stop, or suggesting rules to allow for smoking. People suggested rules for staff members to be allowed to smoke.

*“If staff who smoke choose to smoke in ‘work time’ it should only be in their break/lunch time”*

There is also a sense that an outright ban would be dangerous. Some people raised concerns that if smoking was banned, people would continue to smoke surreptitiously, causing risk within the hospital grounds; in particular a risk of fire from misplaced cigarette stubs.

*“Expecting patients to go off site to smoke is both unrealistic and unsafe”*

These utterances attempt to balance the rights and needs of the smoker with those of the non-smoker. Here participants acknowledged that a number of hospital users smoke and will continue to do so and suggested ways of managing smoking safely and fairly.

### Theme 4: smoke in our space: the battleground

This theme encompasses comments from 65% (n = 178; n = 23 patients, n = 109 staff, n = 42 visitors, n = 4 other) of participants. Utterances revealed an acknowledgement amongst many participants that smokers and non-smokers share one environment, and that participants realise that whether they smoke or not, smoke and smoking behaviours of others encroach into their living space and impact negatively upon the non-smoker.

*“smoke contaminates our impressive atrium area”*

Participants commented on how the hospital entrance was a particular point of contention. Utterances showed how people do not like walking through smoke, smelling smoke, or seeing the mess of cigarettes at the entrance to the hospital.

*“People smoking in the entrance is just awful: the mess they make”*

*“It’s very unpleasant, when leaving the main entrance, to be met with a face full of second hand cigarette smoke”*

Utterances revealed how Pinderfields’ hospital entrance is comparable to a battleground over which the smoker and the non-smoker fight to claim.

Within this theme of smoking and space, people suggested ways to separate and manage smokers. There was support for a smoking shelter or separated area for hospital users to go to in order to smoke.

*“I feel an allocated area should be provided so as not to affect the non-smokers. Also, it looks better when approaching the entrance if it’s not surrounded by people smoking”*

Participants suggested provision of a smoking shelter or designated area for smokers, and justified how a separate space for smokers would improve the current situation. There was a strong sense from n = 147 (54%) participants that the smoking ‘problem’ could be addressed by a separate space for smokers.

### Theme 5: no smoking please!

Participants expressed a view that people shouldn’t smoke and that there should be an outright ban or policy on no smoking. Utterances within this theme reveal how some participants (n = 70, 26%; n = 17 patients, n = 38 staff, n = 13 visitors, n = 2 other) feel that decisive action is needed to stop people smoking.

*“Fine smokers immediately, no excuses”*

*“No smoking should be compulsory and penalties imposed”*

These participants did not attempt to engage with understanding why people smoke, but merely stated that smoking should either be banned, or controlled with methods of enforcement through fines or policing to ensure that smokers adhere to the policy. Although 63% (n = 44) of participants within this theme did not make other comments, there remains 37% (n = 26) whose comments spanned the remaining 4 themes.

## Discussion

Synthesis of this data analysis showed how these themes form a narrative about how hospital users understand smoking within a place that attempts to promote a ‘smoke free’ environment.

The themes all express a sense of agreement that smoking is a social issue. People accept that smoking is detrimental to health and wellbeing. They dislike the smell of smoke and the sight of cigarette ends left on the ground, as well as seeing smokers within a hospital setting. The no-smoking law has perhaps changed people’s acceptance of smokers sharing the non-smokers environment. It appears that most participants acknowledge that smoking is an unhealthy and socially unacceptable behaviour, however their understanding of smokers’ actions and their expressions of how to manage smoking in public spaces vary.

Hospitals have a duty to protect the health and wellbeing of staff, visitors, and patients. Over 12,000 deaths among people over 20 years of age each year are estimated to be attributable to exposure to second-hand smoke [3]. People smoking at the entrance to NHS trusts gives a poor impression, leads to increased litter of cigarettes butts and also means that those entering and leaving buildings have to pass through tobacco smoke. Allowing patients to smoke while in hospital puts them at increased risk of complications and delays their recovery. Having a smokefree policy in a NHS hospital which is not adhered to or enforced undermines public health messages around the dangers of smoking.

Some participants attempt to understand smoking behaviour and accept that smokers have personal reasons for smoking: for example addiction or stress, and try to manage the issue through separation of smokers from non-smokers, helping smokers to quit, and providing time and space for staff to smoke.

Management of space is a prominent theme within the analysis and represents a large number of participants (n = 178, 65%). Proportionally participants in this theme are predominantly staff (n = 109) but all user groups are well represented in this category (i.e. 23 of the 52 patients, 104 of the 141 staff, n = 42 of the n = 74 visitors). This study cannot provide a definitive comment on the expectation that hospital users should have a designated smoking area (e.g. smoking shelter) but it appears that many of participants represented by this theme would support the creation of such an area in order to manage the impact of smoke on the grounds.

We identified a relatively small group of people who took a harder approach and believed that smokers should no longer be allowed to smoke within hospital grounds. They suggest enforcing a law or no smoking policy with policing efforts or fines. Within this theme, ‘no smoking please!’, n = 38 (63% of the 70 participants included in this theme) were categorised solely in this theme. These participants do not reflect on the issue of smoking in further depth, or consider other methods of management. This theme contains the highest number of people who comment within one theme only.

The ‘smokers are free to do as they wish’ theme appears to be an absolute sentiment that it is not appropriate to interfere with another individual’s behaviour, and distinct from the other themes that accept smoking is a social issue that needs controlling. Out of the relatively small number (n = 12, 4%) of participants who commented within this theme, 59% (n = 7) coupled this viewpoint with another utterance in a separate theme. Despite an acceptance and belief in people’s freedom to choose how to behave these participants acknowledged smoking as socially unacceptable, and therefore perceived controlling smoking behaviour to be justified. There was one individual who felt that patients were free to smoke if they wished, but that pregnant women should not be allowed to smoke, showing that participants who believed smokers were free to do as they wish provided caveats to this assertion which determined which individuals had this freedom of choice. The free text section allowed participants to consider their views on smoking and reveals that smoking is a complex social behaviour with a divided opinion.

Our search of the literature revealed limited published evidence that explores people’s perceptions and viewpoints of smoking in and around hospitals with a no smoking policy in depth. Research has looked into changing smoking practice in hospitals with no smoking policies [8]. Research has also explored the difficulties and challenges associated with no smoking policies in hospitals, such as enforcement of policies or maintaining smoke free areas [9, 10]. The current study provides an understanding of how hospital users attempt to make sense of the management of smoking within public spaces, in particular smoking within a health care setting.

This study has highlighted one of the challenges in the implementation and enforcement of NHS Hospitals no smoking policies. The results from this study have provided staff at the hospital the evidence that the majority of people do not want people to smoke in the hospital entrance and believe it should be kept smokefree. This may encourage other people to conform to the ‘norm’ and either not smoke or smoke elsewhere and give staff the confidence to challenge smokers at the entrance knowing that the majority of people agree with keeping the entrance smokefree.

The strengths of this study lie in the projects’ ability to build relationships with hospital management and staff which helped gain access throughout the hospital including to inpatients on the ward. This also helped ensure there was even representation from the three target groups of staff, visitors, and patients. The anonymous nature of the survey aimed to encourage participants to provide honest responses and the information provided in the optional free text box suggests that participants felt comfortable sharing their views with the project staff and did so willingly and without fear of reprisal. The analysis of the free text responses has enabled us to uncover complexity of viewpoints that may not have been apparent had we considered only the quantitative results (results that suggested that 99% of participants do not smoke in hospital entrances). Limitations of this study include the sample being one of convenience rather than randomly or purposively selected. The embedding of a free text response in a structured survey limited the richness of the data collected – and therefore the data lacks the depth one would expect from a traditional qualitative study. The data provided by participants was however unexpectedly relatively rich for a single free text response box and therefore warranted systematic analysis and synthesis. The results suggest merit in future research investigating in more depth the complexities of the viewpoints of hospital patients, staff, and patients towards NHS smokefree policies and their implementation.

## Conclusions

Smoking remains a contentious issue amongst hospital staff, patients and visitors. Despite a no smoking policy, a minority of hospital users continue to smoke within hospital grounds. In addition, there is a perception among hospital users that smokers continue to smoke at the hospital entrance; this despite the vast majority of participants being non-smokers and/or not smoking at the hospital entrance. Participants acknowledge that smoking is an increasingly unacceptable social behaviour but their understanding and acceptance of smokers vary. There is a strong sense of dislike about the impact of smoke and smokers on the shared hospital environment, with a focus on the hospital entrance. Many participants suggest separating smokers and non-smokers and moving smokers away from the hospital entrance with the introduction of smoking shelters.

Policy must account for the impact smoking has upon other people and non-smokers, and upon the space of the hospital grounds which all hospital users share. Methods of managing the impact of smoke on others include use of smoking shelters or total prohibition and enforcement of non-smoking policies throughout hospital grounds. The current study suggests that to gain support of hospital users hospital smoking policy needs to address both the individual’s right to choose to smoke and the social responsibility to control and limit smoking within a health care setting.

## Electronic supplementary material

Additional file 1: **RATS Guidelines.** (DOCX 28 KB)

Additional file 2: **Thematic codes used to classify utterances.** (PDF 34 KB)
