# Supplementary material for: NHS patients, staff, and visitor viewpoints of smoking within a hospitals’ ground: a qualitative analysis
Source: BMC Public Health. 2014 Sep 29;14:1015. doi: 10.1186/1471-2458-14-1015 (PMC4247108; doi:10.1186/1471-2458-14-1015)
Supplement: Supplementary file 1 — Additional file 1: RATS Guidelines. (DOCX 28 KB) [file 12889_2014_7307_MOESM1_ESM.docx]

| **THIS SHOULD BE INCLUDED IN THE MANUSCRIPT** |  |  |
| --- | --- | --- |
| Research question explicitly stated  Research question justified and linked to the existing knowledge base (empirical research, theory, policy) | **Objective:** To explore patient, staff, and visitor viewpoints of smoking within the grounds of a National Health Service hospital.  The introduction positions our study in light of the Smokefree legistlation introducted in England in 2007 and the policies being implemented within NHS services. |  |
| Study design described and justified i.e., why was a particular method (e.g., interviews) chosen? | Design: The current qualitative study is a secondary analysis of free text responses provided by participants to an optional question included as part of a wider questionnaire investigating the attitudes and behaviour of hospital patients, staff, and visitors towards smoking on hospital premised. The original study utilised two cross-sectional survey data collection points and aimed to investigate actual and perceived social norms associated with smoking in hospital entrances and on hospital grounds. |  |
| Criteria for selecting the study sample justified and explained   - *theoretical:* based on preconceived or emergent theory - *purposive:* diversity of opinion - *volunteer:* feasibility, hard-to-reach groups | Secondary analysis of data – sampling strategy of the original study is described. |  |
| Details of how recruitment was conducted and by whom | “Paper surveys were distributed by employees of Magpie Creative Communications throughout the hospital and grounds (e.g. hospital wards, administrative staff areas, canteen areas, hospital shuttle bus queue).” |  |
| Details of who chose not to participate and why | This was an anonymous survey, we have no further detail on the n=37 (4%) surveys that were not returned. Details of surveys excluded are included “Of these 23 were excluded (n=20 age<18 years, n=3 <50% completion).” |  |
| Method(s) outlined and examples given (e.g., interview questions) | “Data collection: The wider study collected data on the difference between the perceived and reported levels of support for smokefree hospital entrances and grounds. The survey title explained to participants that the questions were aimed to understand what people think about smoking around the grounds of Pinderfields Hospital. These questions included one free text box that asked “If you have any other comments you would like to add, please put them here”. “ |  |
| Study group and setting clearly described | “Setting: Pinderfields Hospital is a UK National Health Service hospital based in Wakefield in the county of Yorkshire. It is part of the Mid Yorkshire Hospitals NHS Trust which provides community, acute (hospital-based treatment) and specialist health services to around half a million people living in the Wakefield and North Kirklees area. Surveys were distributed within between 10-18^th^ September and 17^th^-21^st^ December 2012.” |  |
| End of data collection justified and described | Secondary analysis of data. The original data collection in the original study was time limited due to the audit informing an intervention that was to begin in the New Year. |  |
| Do the researchers occupy dual roles (clinician and researcher)? Are the ethics of this discussed? Do the researcher(s) critically examine their own influence on the formulation of the research question, data collection, and interpretation? | Researchers do not occupy dual roles. |  |
| Informed consent process explicitly and clearly detailed |  |  |
| Description of the basis on which quotes were chosen  Semi-quantification when appropriate  Illumination of context and/or meaning, richly detailed | Ethics:  Research and Development approval was received (Mid Yorkshire NHS Trust). Study approved as an audit and evaluation of smoking behaviour on Pinderfields hospital grounds and ethical approval was not required.  All participants gave their permission to be part of the audit and were given pertinent information to make an informed consent to participate. Individuals had the right to refuse to participate if they wished. |  |
| Method of reliability check described and justified e.g., was an audit trail, triangulation, or member checking employed? Did an independent analyst review data and contest themes? How were disagreements resolved? | Analysis: The comments from 306 participants were broken down into 480 utterances with separate meanings. Data were analysed using a thematic analysis. Using an inductive approach, utterances were searched for items of interest and recurrent themes. Codes were iteratively developed with discussions between authors AS and BMB; disagreements were resolved through a process of consensus. The final coding structure enabled categorisation of all utterances. Analysis revealed 23 codes (see Appendix A for list of codes), which were bought together to form 6 themes. |  |
| Findings presented with reference to existing theoretical and empirical literature, and how they contribute | Discssion links results to NHS policy and implications for practice. |  |
| Strengths and limitations explicitly described and discussed | “The strengths of this study lie in the projects’ ability to build relationships with hospital management and staff which helped gain access throughout the hospital including to inpatients on the ward. This also helped ensure there was even representation from the three target groups of staff, visitors, and patients. The anonymous nature of the survey aimed to encourage participants to provide honest responses and the information provided in the optional free text box suggests that participants felt comfortable sharing their views with the project staff and did so willingly and without fear of reprisal. The analysis of the free text responses has enabled us to uncover complexity of viewpoints that may not have been apparent had we considered only the quantitative results (results that suggested that 99% of participants do not smoke in hospital entrances). Limitations of this study include the sample being one of convenience rather than randomly or purposively selected. The embedding of a free text response in a structured survey limited the richness of the data collected – and therefore the data lacks the depth one would expect from a traditional qualitative study. The data provided by participants was however unexpectedly relatively rich for a single free text response box and therefore warranted systematic analysis and synthesis. The results suggest merit in future research investigating in more depth the complexities of the viewpoints of hospital patients, staff, and patients towards NHS smokefree policies and their implementation.” |  |
| Evidence of following guidelines (format, word count)  Detail of methods or additional quotes contained in appendix  Written for a health sciences audience | An appendix of the coding frame is provided.  The articles is written for health sciences audience.  Format and word count have been adhered to. |  |
