# Supplementary material for: NHS patients, staff, and visitor viewpoints of smoking within a hospitals’ ground: a qualitative analysis
Source: BMC Public Health. 2014 Sep 29;14:1015. doi: 10.1186/1471-2458-14-1015 (PMC4247108; doi:10.1186/1471-2458-14-1015)
Supplement: Supplementary file 2 — Additional file 2: Thematic codes used to classify utterances. (PDF 34 KB) [file 12889_2014_7307_MOESM2_ESM.pdf]

| | CODE | DESCRIPTION/DEFINITION | THEMES |
|---|---|---|---|
| 1 | Acceptance of smokers ( no condition) | Not getting involved in what smokers do and allowing smokers to be smokers, or acceptance that people smoke. Sentiment that it isn't appropriate to get involved in other people's behaviour. e.g. talk of 'human rights', 'freedom to smoke', legality of smoking | 2: Smokers are free to do as they wish |
| 2 | Justification of why people smoke | Acknowledging hospitals as environments where people are stressed or upset, and smoking is a way of dealing with this, that it is an addiction, and hard to give up. Sentiment that because of stressors, smokers should not be judged, and allowed to have the space/right to smoke | 3: The poor smoker |
| 3 | No smoking outright | Ideas to blanket ban smoking in and around hospitals, that if smoking is bad/unhealthy/an issue, just to ban it completely. This can be linked to ways of enforcing this ban | 5: No smoking please |
| 4 | Methods for enforcing no smoking | Suggestion to enforce no smoking, or rules of smoking through fines and financial consequences, or monitoring, hospital policy or changing the law. Also includes methods to prevent people smoking, such as patches- regardless of feasibility of this! | 5: No smoking please |
| 5 | Health promotion | Suggestions to help people stop smoking: through various methods (leaflets/encouragement/electric cigarettes) | 3: The poor smoker |
| 6 | Have signs | Suggestions to have signs to guide people: on where to smoke, or where not to smoke | 4: smoke in our space |
| 7 | People will smoke, despite rules or any idea suggested | Noticing how or why smokers or people generally ignore the rules, signs, bins etc. Sentiment that stopping smoking is too difficult or complicated due to how hard it is to enforce. Acknowledgement of difficulty to enforce stopping or expression of doubt that this is a manageable issue | 1: Smoking is a dirty problem |
| 8 | People shouldn't smoke | Comments that people shouldn't smoke at all either generally or in context of hospital setting, with or without explanations why, and that people shouldn't be allowed to smoke: but no specific way on enforcement. Sentiment that it is unhealthy, unwise, unsafe, and wrong | 5: No smoking please |
| 10 | Smokers not conforming to social norms | Comment on seeing staff, sick people, patients smoking, and disbelief. Ideas that people should respect rules if rules are in place. Sentiment that it looks bad, either personally, or a poor reflection on the hospital, or that doesn't help people wanting to give up. Hospitals are a place of health and health promotion, so they should show this sentiment pubicly. | 1: Smoking is a dirty problem |
| 11 | Impact of smoking on surroundings | Comments on the mess or unsightlines of cigarettes, on the smell or sight of smoke drifting into areas that affect other people around the hospital and how others are negatively affected by smokers behaviour, walking through smoke etc | 4: smoke in our space |
| 12 | Non smokers have rights too | Comment on how smokers *should* not impact on non smokers, or others, including staff in hospital, an element of rights or implicit demand from non smokers that their health is important | 1: Smoking is a dirty problem |
| 13 | Management of smokers area | People who acknowledge a problem and suggest ways of separating smokers and non smokers through use of area: away from entrance, designated space or shelter | 4: smoke in our space |
| 14 | Benefits of management of smokers space | Improvements to be made by having a separate space for smokers: cleaner etc, tidier, better | 4: smoke in our space |
| 15 | Space for staff and space for patient smokers | Acknowledgement of a problem and a way to manage or control this through space but with a focus on keeping staff and patients separate | 4: smoke in our space |
| 16 | Need for safety | Allowing people to smoke in a safe environment within hospital grounds, an acknowledgment that smoking off site, or in a deregulated way may be 'dangerous' | 3: The poor smoker |
| 17 | Ideas of infection | Comments on how leaving hospital to smoke and returning brings infection risk to the hospital | 1: Smoking is a dirty problem |
| 18 | Design and question technicalities | Comments on survey technicalities: questions inappropriately laid out or unclear | 6: Misc |
| 19 | Why this survey is not useful | Comments on why the survey is useless: not asking the right things, or waste of money/time/resources. Other issues to consider | 6: Misc |
| 20 | Staff rules | Sentiment that there should be rules for staff smokers: smoking on breaks only, changing clothes, being 'fair' for staff non smokers | 3: The poor smoker |
| 21 | Need for change/control | Acknowledgment that something needs doing on the smoking issue, that it is a widespread problem, with doubt or no specifics on how to achieve change, or an expression of a need to control the issue that is not related to a smokers area | 1: Smoking is a dirty problem |
| 22 | Misc | Miscellaneous: brief utterances with no meaning | 6: misc |
| 23 | Misc with meaning | Miscellaneous: utterances with meaning but which focus only on process/content of the project | 6:misc |
